# Implementation research to catalyze advances in health systems strengthening in sub-Saharan Africa: the African Health Initiative

**DOI:** 10.1186/1472-6963-13-S2-S1

**Published:** 2013-05-31

**Authors:** Kenneth Sherr, Jennifer Harris Requejo, Paulin Basinga

**Affiliations:** 1 Department of Global Health, University of Washington, Seattle, WA, USA; 2 Health Alliance International, Beira, Mozambique; 3 Department of International Health, The Johns Hopkins Bloomberg School of Public Health, USA; 4 National University of Rwanda, School of Public Health, Rwanda; 5 The Bill and Melinda Gates Foundation, Seattle, WA, USA

## 

The importance of strengthening health systems has gained increased attention in recent years, and there have been renewed calls for a focus on health systems as part and parcel of meeting the health related Millennium Development Goals.[1,2] Despite the growing focus on health systems, the largest global health initiatives – such as PEPFAR, PMI, the Global Fund to Fight AIDS, TB, and Malaria, and GAVI – continue to have a disease specific focus. The divergence in opinion on what constitutes health systems strengthening and the scarcity of rigorous evaluations of various approaches undermine efforts to focus on health systems as a means of improving population health. In response to this challenge, the Doris Duke Charitable Foundation (DDCF) launched the African Health Initiative (AHI) to catalyze significant advances in strengthening health systems by supporting Population Health and Implementation Training (PHIT) Partnerships in five diverse sub-Saharan African contexts. Each Partnership is implementing and evaluating an innovative project designed to address key health systems constraints and improve service delivery and health outcomes.

As part of a larger aim of fostering global learning through the Partnership activities, a PHIT Data Collaborative with representation from all key partners involved in the AHI was established to stimulate cross-site research, timely dissemination of findings, and use of results for programming purposes, as well as policy and strategy formulation. The Data Collaborative is managed by a Collaborative Management Committee (CMC) that is comprised of one voting representative of the co-PIs of each PHIT Partnership and the Data Coordinator (a small team based at Johns Hopkins University responsible for developing an evaluation framework for the Collaborative and to provide technical support as needed to the teams and the foundation), as well as a non-voting member from the DDCF.[3] The foundation created a modest fund to support cross-project activities identified by the CMC.

Annual meetings of the PHIT Data Collaborative are held to review progress with PHIT Partnership activities over the course of the year, provide ongoing updates on implementation, enable partnership teams to present and discuss initial results, as well as prioritize and select cross-Partnership activities to be supported through the CMC funds.

At the first Global Health System Symposium held in Montreux, Switzerland in November 2010, the AHI CMC organized a round table to present the final Partnership designs. Round table participants requested ongoing progress updates, and recommended that early experiences be disseminated broadly to inform similar activities in the field. The idea for this supplement was generated at the second annual PHIT Data Collaborative meeting in Ifakara, Tanzania in October, 2011. Throughout the third year of PHIT Partnership implementation, the CMC defined the scope of the supplement, agreed on manuscript topics, developed a standardized outline for country design papers, and developed a timeline for the development and dissemination of the supplement. In June, 2012, two representatives from each PHIT Partnership and the Data Coordinator, together with DDCF staff, participated in a one-week writing workshop in Baltimore, MD, to develop, review, critique, and improve first drafts of the supplement articles.

The supplement that resulted from these efforts showcases what has been learned in the first three years of the AHI. The first paper [4] describes the history of the AHI and the five selected Partnerships, places the project in the context of broader discussions on the added value of health systems strengthening and implementation research, and presents notable lessons learned from the AHI to date.

The next five papers [5-9] describe each PHIT Partnership’s implementation strategy and evaluation design, and how the project evolved during the initial two years of implementation. These descriptions reflect the different experiences and perspectives of the implementing institutions that form each Partnership. For example, the Ghana and Tanzania Partnerships approached health systems strengthening from a surveillance lens, and build on the experiences of the Tanzania Essential Health Intervention Project (TEHIP) and Ghana’s Community-based Health and Planning Services project (CHPS). The Mozambique experience builds on a partnership that has supported service scale-up for over 25-years through broad integration into the public sector’s Primary Health Care framework. The Rwandan experience builds on a grassroots development foundation, with a strong commitment to engagement with both community and health systems structures. Finally, the Zambian Partnership approached health systems strengthening from its experience designing and implementing large scale HIV programs with a focus on the clinician-patient relationship. Despite their varied origins and perspectives, all five projects are united by their commitment to large scale health systems strengthening, and rigorously evaluating its impact on service delivery and population health.

The design papers are followed by two papers on cross-cutting areas important to all five of the Partnership teams: approaches to improving health information systems and data for decision making [10], and improving service quality and its measurement [11]. The last paper in the supplement introduces the AHI common evaluation framework [12], including core and common measures and the process for their selection, and a documentation component.

The supplement concludes with a set of commentaries by four experts [13-17] that reflect on the challenges inherent in this type of work, the potential of rigorous qualitative and quantitative implementation research to bridge the ‘knowledge to action’ gap, and the expected contribution from the AHI.

Although PHIT Partnership implementation is still underway, this supplement identifies a number of overarching lessons from the first three and a half years of implementation that will be of interest to multiple readers from the policy setting, donor, implementer, and health system research communities. These include the need for a multipronged approach to systems, with the result that most of the teams ultimately included activities in each of six areas identified as health system building blocks by the World Health Organization. Although country institutions are not a direct recipient of funds, the Foundation required written support from Ministries of Health as part of an explicit effort to assure country ownership. Despite relatively modest funds for the scope of planned activities – especially when compared to large, countrywide global health initiatives such as the Global Fund or PEPFAR – teams garnered substantial interest and support at high levels of the Ministries of Health, reflective at least in part of the large perceived need by countries to plan comprehensively for health systems without the constraint of a single disease focus. This support was instrumental to the ability of the teams to successfully get their projects underway. Funding beyond the usual two to three year grant cycle was greeted with enthusiasm by both grantees and Ministries, but the challenge of maintaining continuity in personnel over the five to seven year implementation period affected all Partnership teams. In some cases, the leadership of teams experienced complete turnover, and all teams have experienced change in senior program management. Finally, although all PHIT Partnership teams agreed that achieving a mortality reduction within 4 years of full intervention implementation was an ambitious goal, there has been considerable impatience in waiting for results to answer “whether it worked.”

As the AHI Partnerships wind down over the next two and a half years, and the results from the AHI impact evaluation become available, a wealth of knowledge will be generated on if, how, and why (or why not) the individual Partnership strategies strengthened health systems, improved the delivery of integrated primary care, and led to measurable improvements in population health. When viewed collectively, the results of this evaluation will provide a rich understanding of the commonalities and differences in health system strengthening approaches tested across the Partnerships, their feasibility, applicability, “scalability,” and impact. It is expected that a follow-up series will be published at the end of the project implementation period that will present the final outcomes and impact of the PHIT projects.

The lessons learned from the AHI are especially salient given the crossroads we face today. As the global health community anticipates the 2015 Millennium Development Goals deadline and looks forward to building consensus for future priorities, [18] and as PEPFAR reflects on the results of its second five-year evaluation,[19] it is clear that strengthening health systems will continue to be a central strategy for achieving healthy life expectancy and universal access to quality health services in the years to come. By implementing and testing novel approaches to improving population health by strengthening and integrating primary health care, the AHI is well placed to contribute to this discourse.

## Competing interests

The authors declare that they have no competing interests.

## Declarations

This article has been published as part of *BMC Health Services Research* Volume 13 Supplement 2, 2013: Improving primary health care to achieve population impact: the African Health Initiative. The full contents of the supplement are available online at http://www.biomedcentral.com/bmchealthservres/supplements/13/S2. Publication of this supplement was supported by the African Health Initiative of the Doris Duke Charitable Foundation.
